# Optimal management of renal cell carcinoma in octogenarians: Retrospective analysis using updated Korean Renal Cell Carcinoma (KORCC) database

**DOI:** 10.1371/journal.pone.0283483

**Published:** 2023-03-30

**Authors:** Jung Kwon Kim, Gyoohwan Jung, Cheol Kwak, Chang Wook Jeong, Seok Ho Kang, Sung-Hoo Hong, Yong-June Kim, Jinsoo Chung, Eu Chang Hwang, Tae Gyun Kwon, Seok-Soo Byun

**Affiliations:** 1 Department of Urology, Seoul National University Bundang Hospital, Seongnam, Korea; 2 Department of Urology, Seoul National University College of Medicine, Seoul, Korea; 3 Department of Urology, Hanyang University Seoul Hospital, Seoul, Korea; 4 Department of Urology, Seoul National University Hospital, Seoul, Korea; 5 Department of Urology, Korea University Anam Hospital, Seoul, Korea; 6 Department of Urology, Seoul St. Mary’s Hospital, College of Medicine, The Catholic University of Korea, Seoul, Korea; 7 Department of Urology, Chungbuk National University Hospital, Cheongju, Korea; 8 Department of Urology, Center for Prostate Cancer, National Cancer Center, Goyang, Korea; 9 Department of Urology, Chonnam National University Medical School, Gwangju, Korea; 10 Department of Urology, Kyungpook National University Chilgok Hospital, Daegu, Korea; 11 Department of Medical Device Development, Seoul National University College of Medicine, Seoul, Korea; Ajou University School of Medicine, REPUBLIC OF KOREA

## Abstract

**Background:**

There is few of optimal management guideline in elderly patients with renal cell carcinoma (RCC). To compare the survival outcomes of octogenarian RCC group and younger RCC group after surgery using nationwide multi-institutional database.

**Methods:**

A total of 10,068 patients who underwent surgery for RCC were included in the current retrospective, multi-institutional study. A propensity score matching (PSM) analysis was conducted to control other confounding factors in analyzing survival outcomes of octogenarian and younger group RCCs. Kaplan-Meier curve analysis to calculate the survival estimates for cancer-specific survival (CSS) and overall survival (OS), and multivariate Cox-proportional hazard regression analyses to evaluate the significant variables associated with the survival outcomes were also performed.

**Results:**

Both groups were well-balanced in all baseline characteristics. In a total cohort, Kaplan-Meier survival analysis showed a significantly decreased 5-year and 8-year CSS and OS in the octogenarian group compared with the younger group. However, in a PSM cohort, no significant differences were evident between the two groups in terms of CSS (5-year, 87.3% vs. 87.0%; 8-year, 82.2% vs. 78.9%, respectively, log-rank test, p = 0.964). In addition, age ≥ 80 years (HR, 1.199; 95% CI, 0.497–2.896, p = 0.686) was not a significant prognostic factor of CSS in a PSM cohort.

**Conclusions:**

The octogenarian RCC group after surgery had comparable survival outcomes compared with younger group after PSM analysis. For the life expectancy of octogenarian is getting longer, active treatment is considerable in patients with good performance status.

## Introduction

The incidence of renal cell carcinoma (RCC) is increasing each year [1]. In Korea, according to the latest cancer incidence statistics from the Korea Central Cancer Registry, there were 6026 cases of kidney cancer in 2019 [2]. RCC occurs predominantly in the sixth to eighth decade of life, according to the 2003 to 2007 National Cancer Institute Surveillance, Epidemiology and End Results (SEER) Cancer Statistics Review; it is unusual in patients under 40 years of age and rare in children [3–5]. Along with an increasing average life expectancy and broader adoption of imaging work-up, the incidence of RCC in elderly patients older than 80 years has increased. Notably, based on data from the KOrean Statistical Information Service (KOSIS), 80-year-old women and men have median remaining life expectancy of 10.7 and 8.4 years in 2019, respectively [6]. Thus, the need for patient counseling regarding optimal treatment in this age group is also increasing.

RCC in elderly patients is often known to have more aggressive features [7–10]. Using the data of 59,944 RCC patients who underwent surgery in SEER database, Hellenthal et al. [9] found that octogenarian group were 2.32 times more likely to die (95% confidence interval [CI], 2.22–2.42, p < 0.001) and 1.33 times more likely to die of RCC (95% CI, 1.23–1.43, p < 0.001) than younger group. Additionally, in octogenarian group, patients who underwent radical nephrectomy (RN) were 2.54 times more likely to die of RCC (95% CI 1.68–3.84, p<0.001) than partial nephrectomy (PN). Similarly, using the data of 6,234 patients in European multinational database (Collaborative Research on Renal Neoplasms Association [CORONA]), May et al. [10] also found that octogenarians had unfavorable outcome regarding cancer specific mortality (hazard ratio [HR], 1.48; p = 0.042) and overall mortality (HR, 4.32; p<0.001) compared with younger patients with RCC and differ significantly from these in clinical parameters.

Although surgery, especially PN, is considered the mainstay of treatment for localized RCC, current guidelines and treatment protocols have been developed primarily based on patients with typical disease-onset age and longer life expectancy [11]. It is well-known that PN provides oncologic equivalency to RN while preserving kidney function and potentially preventing non-oncologic morbidity and mortality [12, 13]. However, according to a previous reported meta-analysis [14], PN had been rarely performed in elderly patients; instead, active surveillance (AS) was more prevalent especially for small renal masses.

Unfortunately, there is few of optimal management guideline in elderly patients with RCC. In addition, large cohort studies investigating survival outcomes in this group are still lacking in Asian population. Thus, we compared the survival outcomes of octogenarian RCC group and younger RCC group after surgery using nationwide multi-institutional database in Korea. A comparative analysis between the patients who underwent PN versus RN was also performed in octogenarian RCC group.

## Materials and methods

### Ethics statement

We performed this retrospective, multi-institutional study after Institutional Review Board (IRB) approval of Seoul National University Bundang Hospital (Approval number: B-2202-739-101) and in accordance with the ethical standards of the 1964 Declaration of Helsinki and its later amendments. The waiver of the informed consent requirement was approved by the IRB considering the retrospective study design involving anonymized data. Personal identifiers were completely removed and the data were analyzed anonymously.

### Study cohort

We used the Korean Renal Cell Carcinoma (KORCC) database which was first established in 2011 and comprises 8 academic institutions nationwide [15]. Recently, data of each institution were updated from March to June 2021. Subsequently, the updated KORCC database covered data on a total of 10,068 patients who have received surgery for RCC. All institutions obtained institutional review board approvals before inputting data into the database. Unified data templates were used for consistent data collection at each institution. Survival data were retrospectively reviewed from medical records or identified from death certificate data.

### Acquisition and definition of data

Clinical perioperative variables included age at surgery, gender, body mass index (BMI), Eastern Cooperative Oncology Group (ECOG) performance status, and types of surgery (partial nephrectomy [PN] vs. radical nephrectomy [RN]), laboratory test (hemoglobin, creatinine, glomerular filtration rate, etc.), estimated blood loss (EBL), and operative time (S1 Fig, S1 File). Cases that underwent cytoreductive nephrectomy for metastasis were excluded. Pathological parameters including pathologic tumor size based on the maximum diameter, pathologic tumor stage according to the 2010 TNM classification systems, and Fuhrmann nuclear grade were also evaluated. Recurrence was defined as radiographically verified distant metastasis or local disease recurrence during the study period. The cause of death was determined by the responsible physicians and death certificates. Overall survival (OS) was calculated from the date of surgery to the date of last follow-up or death. Cancer-specific survival (CSS) was defined as the time between surgery and death due to RCC, or the date of last follow-up. Patients who were event-free at the time of data analysis were censored by the date they were last known to be alive and event-free. Patients without documented death at the time of data analysis were censored as alive on the date of last follow-up.

### Statistical analysis

Clinicopathological characteristics were compared between octogenarian versus younger RCC groups using chi-squared test for categorical variables, and independent t-test for continuous variables. Kaplan-Meier curve analysis was used to calculate the survival estimates for CSS and OS. Further, the log-rank test was used to conduct comparisons between the groups. Univariate and multivariate Cox-proportional hazard regression analyses were performed to evaluate the significant variables associated with the survival outcomes. In addition, we used the propensity score methodology [16] to control other confounding factors in analyzing survival outcomes of octogenarian and younger group RCCs. The propensity score matching (PSM) analysis was conducted using a nonparsimonious multiple logistic regression models, with a 1:1 matching ratio. Subgroup analysis in patients over 60 years was also performed. All statistical analyses were performed using commercially available software (IBM SPSS Statistics ver. 21.0, Armonk, NY, USA and the statistical package for R, ver. 2.13.2, R Foundation for Statistical Computing [http://www.r-project.org/]) and two-sided p values < 0.05 were considered statistically significant.

## Results

### Comparison of clinicopathological features: Propensity score matching analysis

Results of the comparative analysis of clinicopathological features between the octogenarian versus younger RCC group are summarized in Table 1. The propensity scores were computed by logistic regression modeling, with the independent variables showing significant differences between the two groups in comparative analyses: gender, BMI, ECOG, types of surgery, preoperative laboratory test including hemoglobin and glomerular filtration rate, Maximal tumor diameter, Fuhrmann nuclear grade, and follow-up duration. Consequently, the two groups were well balanced except for the age at surgery (mean, 60.7 vs. 82.4 months, p < 0.001). In the matching cohort, there were 39 (20.0%) overall mortalities in the octogenarian RCC group and 41 (21.0%) in the younger RCC group, and 17 (8.7%) and 28 (14.4%) cancer-specific mortalities.

**Table 1:** Baseline characteristics.

	Total cohort (N = 10,068)			Propensity score matching with 1:1 ratio		
Variables	< 80 yrs (N = 9873)	≥ 80 yrs (N = 195)	P	< 80 yrs (N = 195)	≥ 80 yrs (N = 195)	P
Age at surgery, yrs, mean (SD)	61.8 (12.1)	82.4 (2.4)	<0.001	60.7 (11.8)	82.4 (2.4)	<0.001
Gender, n (%)			<0.001			0.416
Male	6826 (69.1%)	110 (56.4%)		101 (51.8%)	110 (56.4%)	
Female	3047 (30.9%)	85 (43.6%)		94 (48.2%)	85 (43.6%)	
BMI, kg/m^2^, mean (SD)	24.5 (3.3)	24.1 (3.2)	0.056	24.0 (3.2)	24.1 (3.2)	0.729
Smoking, n (%)*			<0.001			0.594
Non-smoker	5107 (51.7%)	143 (73.3%)		138 (70.8%)	143 (73.3%)	
Ex-smoker	1499 (15.2%)	23 (11.8%)		20 (10.3%)	23 (11.8%)	
Current-smoker	1392 (14.1%)	16 (8.2%)		24 (12.3%)	16 (8.2%)	
Unknown	1875 (19.0%)	13 (6.7%)		13 (6.7%)	13 (6.7%)	
ECOG, mean (SD)			0.003			0.217
0	6023 (61.0%)	84 (43.1%)		89 (45.6%)	84 (43.1%)	
≥ 1	3850 (39.0%)	111 (56.9%)		106 (54.4%)	111 (56.9%)	
Type of surgery			0.001			0.344
Radical, n (%)	5279 (53.5%)	129 (66.2%)		119 (61.0%)	129 (66.2%)	
Partial, n (%)	4594 (46.5%)	66 (33.8%)		76 (39.0%)	66 (33.8%)	
Surgical approach*			0.164			0.037
Open, n (%)	3984 (40.4%)	64 (32.8%)		84 (43.1%)	64 (32.8%)	
Laparoscopic or Robotic, n (%)	5889 (59.6%)	131 (67.2%)		111 (56.9%)	131 (67.2%)	
Preoperative laboratory test						
Hb, mean (SD)	13.6 (1.9)	12.5 (1.6)	<0.001	12.4 (1.8)	12.5 (1.6)	0.804
Cr, mean (SD)	1.09 (1.14)	1.12 (0.87)	0.595	1.09 (0.48)	1.12 (0.87)	0.698
GFR (MDRD), mean (SD)	80.4 (28.8)	66.5 (23.2)	<0.001	67.4 (21.3)	66.5 (23.2)	0.680
EBL, ml, mean (SD)	300.3 (459.5)	308.3 (335.0)	0.821	334.5 (658.1)	308.3 (335.0)	0.625
Operative time, min, mean (SD)	171.0 (72.1)	167.3 (72.9)	0.484	163.0 (73.3)	167.3 (72.9)	0.568
Maximal tumor diameter, mm, mean (SD)	41.7 (31.0)	46.1 (31.2)	0.071	43.6 (33.1)	46.1 (31.2)	0.494
Fuhrman grade, n (%)			<0.001			0.581
G1-2	5104 (51.7)	72 (36.9)		76 (39.0%)	72 (36.9)	
G3-4	4769 (48.3)	123 (63.1)		119 (61.0%)	123 (63.1)	
Pathologic T stage, n (%)			0.942			0.101
T1-2	6881 (69.7)	137 (70.2)		144 (73.8%)	137 (70.2)	
T3-4	2992 (30.3)	58 (29.8)		51 (26.2%)	58 (29.8)	
Pathologic N stage, n (%)			0.357			0.412
N0/X	9675 (98.0)	188 (96.4)		190 (97.4%)	188 (96.4)	
N1	198 (2.0)	7 (3.6)		5 (2.6%)	7 (3.6)	
Follow-up, years, mean (SD)	61.0 (44.9)	41.3 (38.4)	0.015	42.0 (32.9)	41.3 (38.4)	0.415
Recurrence, n (%)	1273 (12.9)	24 (12.3)	0.914	23 (11.8%)	24 (12.3)	0.914
Cancer-specific mortality, n (%)	640 (6.5)	17 (8.7)	0.238	28 (14.4%)	17 (8.7%)	0.238
Overall mortality, n (%)	1125 (11.4)	39 (20.0)	<0.001	41 (21.0%)	39 (20.0%)	0.900

SD, standard deviation; BMI, Body Mass Index; ECOG, Eastern Cooperative Oncology Group; Hb, Hemoglobin; Cr, Creatinine; GFR, Glomerular Filtration Rate; MDRD, Modification of Diet in Renal Disease; EBL, Estimated Blood Loss

* These variables were not applied to matching.

### Comparison of survival outcomes according to the age

**Fig 1:**
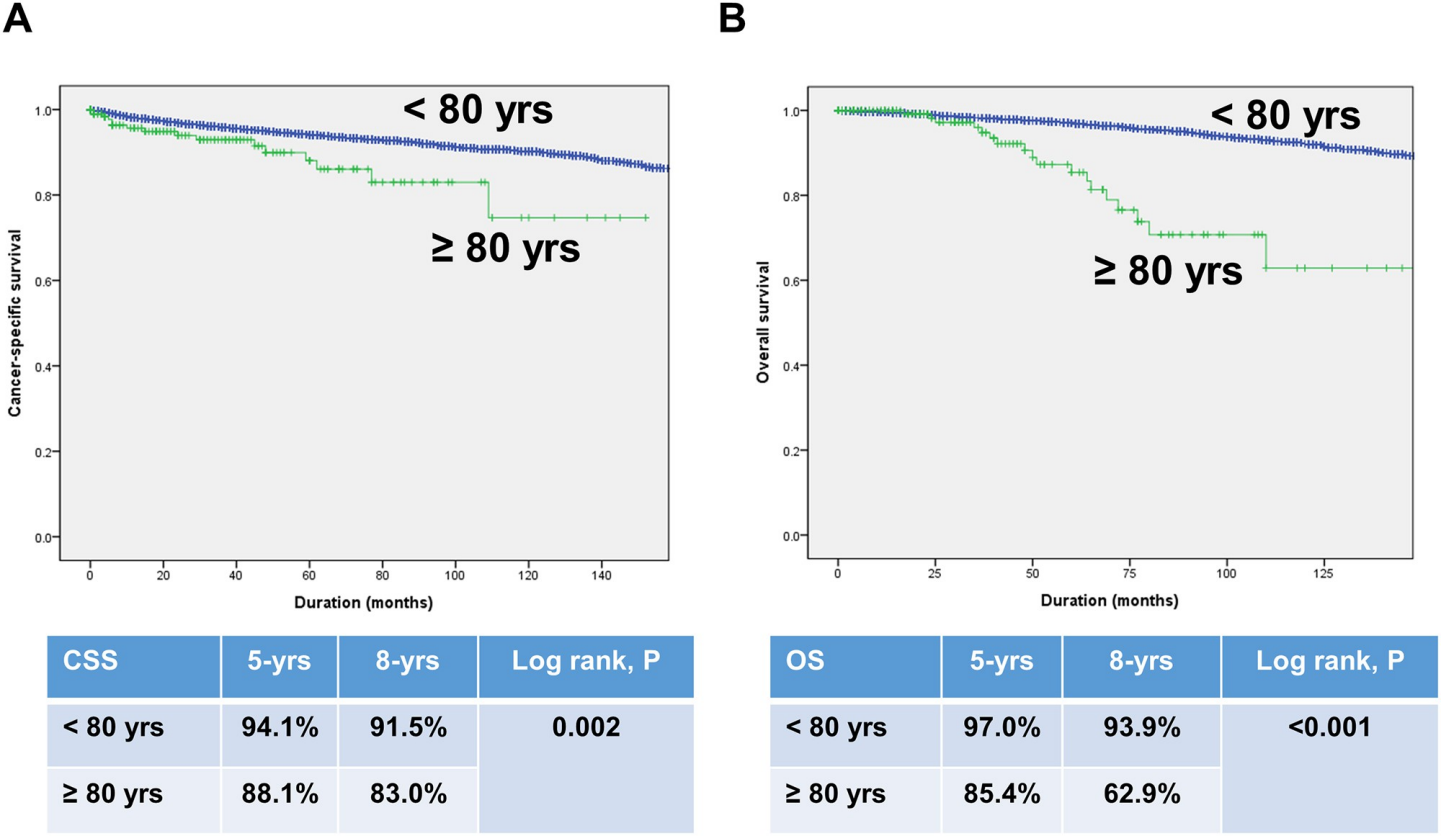
Kaplan-Meier curves for cancer-specific survival (A) and overall survival (B) in patients by age group (octogenarian vs. younger group).

**Fig 2:**
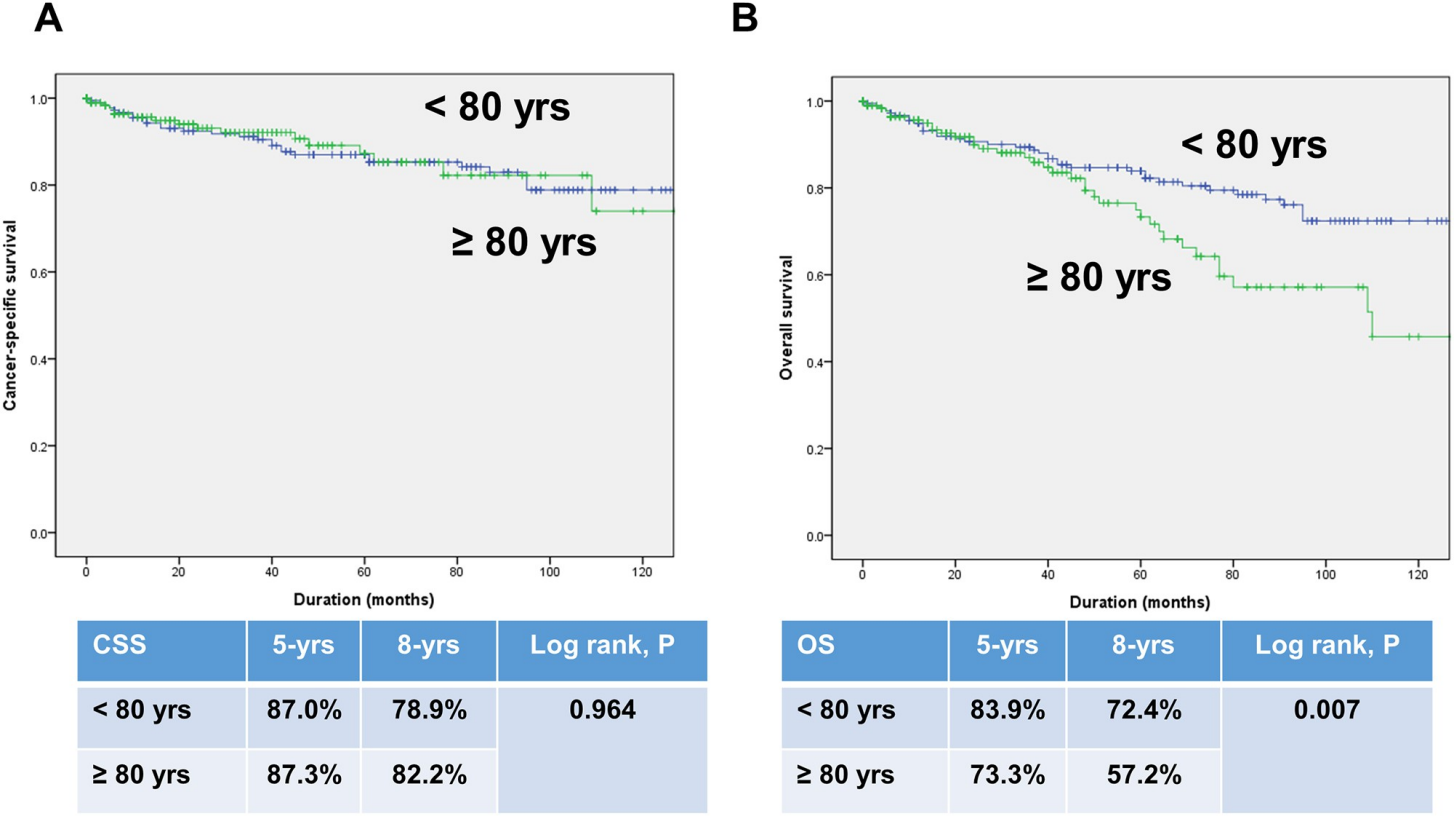
Kaplan-Meier curves for cancer-specific survival (A) and overall survival (B) in patients by age group (octogenarian vs. younger group) after propensity score matching.

In a total cohort, Kaplan-Meier survival analysis showed a significantly decreased 5-year and 8-year CSS (88.1% vs. 94.1%, 83.0% vs. 91.5%, respectively, log-rank test, p = 0.002) and OS (85.4% vs. 97.0%, 62.9% vs. 93.9%, respectively, log-rank test, p<0.001) in the octogenarian RCC group compared with the younger RCC group (Fig 1). In a PSM cohort, Kaplan-Meier survival analysis also showed a significantly decreased 5-year and 8-year OS (73.3% vs. 83.9%, 57.2% vs. 72.4%, respectively, log-rank test, p = 0.007, Fig 2B). However, no significant differences were evident between the two groups in terms of CSS (87.3% vs. 87.0%, 82.2% vs. 78.9%, respectively, log-rank test, p = 0.964, Fig 2A).

We also performed subgroup analysis in patients over 60 years to reduce confounding from unmeasured differences by age. Consequently, we found no significant differences between the two groups in terms of 5-year and 8- year CSS (87.3% vs. 86.5%, 82.2% vs. 76.3%, respectively, log-rank test, p = 0.867, S2A Fig) and OS (73.3% vs. 80.9%, 57.2% vs. 64.3%, respectively, log-rank test, p = 0.110, S2B Fig).

### Comparison of survival outcomes according to the surgery type in octogenarian patients

**Fig 3:**
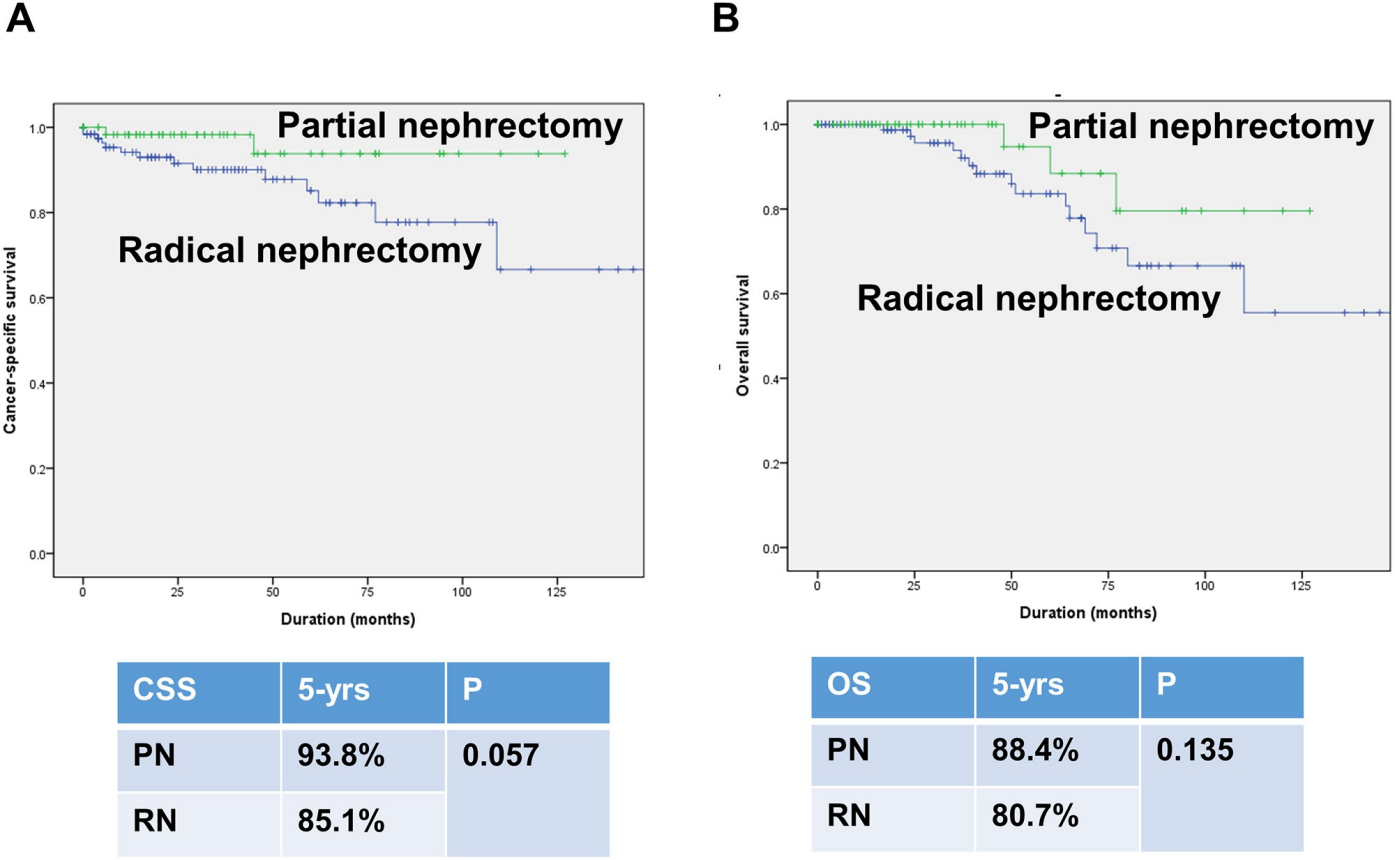
Kaplan-Meier curves for cancer-specific survival (A) and overall survival (B) in octogenarians according to the surgery type.

S1 Table summarized baseline characteristics of octogenarians according to the surgery type. Kaplan-Meier survival analysis showed a decreased 5-year CSS (85.1% vs. 93.8%, log-rank test, p = 0.057) and OS (80.7% vs. 88.4%, log-rank test, p = 0.135) in PN group, albeit the result was not significant (Fig 3).

### Prognostic factors for survival

S2 Table summarized the results of multivariate Cox regression analyses for CSS and OS based on the total cohort. Multivariate analysis revealed that age ≥ 80 years (hazard ratio [HR], 2.385; 95% CI, 1.243–4.580, p = 0.009) was significant prognostic factor of CSS. The other significant prognostic factors for CSS were BMI, GFR (MDRD), type of surgery, pathologic T stage, pathologic N stage, Fuhrman grade, sarcomatoid differentiation, and necrosis (all, p<0.05). In regard of OS, age ≥ 80 years (HR, 3.153; 95% CI, 2.026–4.909, p<0.001) was also significant prognostic factor. Other variables including BMI, GFR (MDRD), female gender, type of surgery, pathologic T stage, pathologic N stage, Fuhrman grade, sarcomatoid differentiation, and necrosis were also significant prognostic factor of OS (all, p<0.05).

However, in a PSM cohort, age ≥ 80 years (HR, 1.199; 95% CI, 0.497–2.896, p = 0.686) was not a significant prognostic factor of CSS (Table 2). Instead, it was still found to be a significant prognostic factor of OS (HR, 1.090; 95% CI, 1.014–3.597, p = 0.045, Table 2). Multivariate analysis revealed that other variables including type of surgery, pathologic N stage, sarcomatoid differentiation, and necrosis were significant prognostic factor of both CSS and OS (all, P<0.05, Table 2).

**Table 2:** Multivariable Cox regression analyses for cancer-specific and other-cause mortality after propensity score matching.

Variables	Cancer-specific mortality HR (95% CI)	P value	Other-cause mortality HR (95% CI)	P value
Age ≥ 80 yrs	1.199 (0.497–2.896)	0.686	1.090 (1.014–3.597)	0.045
BMI	0.963 (0.817–1.135)	0.652	0.945 (0.835–1.069)	0.368
GFR (MDRD)	0.990 (0.967–1.012)	0.367	0.988 (0.970–1.005)	0.159
Gender, female	0.658 (0.278–1.556)	0.340	0.682 (0.353–1.315)	0.253
Type of surgery				
Radical nephrectomy	References		References	
Partial nephrectomy	0.165 (0.037–0.732)	0.018	0.368 (0.165–0.820)	0.014
Maximal tumor size	1.008 (0.996–1.020)	0.171	1.007 (0.997–1.017)	0.167
Pathologic T stage				
T1-2	References		References	
T3-4	1.294 (0.492–3.402)	0.601	1.279 (0.598–2.734)	0.526
Pathologic N stage				
N0/X	References		References	
N1	62.526 (11.028–354.512)	<0.001	30.701 (6.878–137.047)	<0.001
Fuhrman grade				
G1-2	References		References	
G3-4	1.591 (0.462–5.482)	0.462	1.366 (0.625–2.988)	0.434
Sarcomatoid differentiation	7.115 (2.517–20.114)	<0.001	6.708 (2.591–17.366)	<0.001
Necrosis	5.173 (1.854–14.431)	0.002	2.807 (1.197–6.582)	0.018

BMI, Body Mass Index; GFR, Glomerular Filtration Rate; MDRD, Modification of Diet in Renal Disease; HR, Hazard Ratio; CI, Confidence Interval; HR

## Discussion

The present study is based on one of the largest patient groups over 10,000 outside population-based cohorts in Asia, and it represents the first attempt to reliably risk stratify octogenarian patients based on PSM analysis. The vast majority of studies regarding this topic are underpowered and limited by the lack of comparison between octogenarian and an adequate reference patients group [7–10]. Generally, it was suggested that octogenarian patients had been rarely treated with PN and showed lower CSS than younger group. In current study, we also found a significantly decreased 5-year and 8-year CSS and OS in the octogenarian RCC group compared with the younger RCC group in a total cohort. However, after a PSM, no significant differences were evident between the two groups in terms of CSS, even with significant differences in OS. In addition, multivariate analysis also revealed that age (≥ 80 years) was not a significant prognostic factor of CSS. These conflicting results might be reflecting given strong selection biases and confounding factors in retrospective analyses even with a large cohort of patients [9, 10]. PSM controls insufficient number of cases in exact matching by reducing multidimensional to one-dimensional scores, thereby contributing to reducing bias by combining samples with balanced confounders across groups, which can relate to selection bias. In a subgroup analysis of patients over 60 years for more reliable risk stratification, we also found no significant differences between the two groups in terms of 5-year and 8-year CSS and OS. In agreement with our results, Thompson et al. [4] found no significant difference in CSS according to age (p = 0.17) at a median 2.6 years follow-up Using the data of 1,720 patients 18 to 79 years old. Similarly, a French study analyzing 148 octogenarian patients showed a fraction of 10.1% metastatic patients (vs. 14.2% in patients < 80 years, p = 0.022) [8].

For elderly localized RCC patients with limited life expectancy, the role of surgery (RN or PN) over AS remains unclear [17, 18]. In a previous study, Lane et al. [17] confirmed the importance of AS; they found no significant differences in survival outcomes between surgery (PN or RN) and AS among elderly patients with clinically localized RCC. Using instrumental variable analysis with a total of 10,595 patients, Sun et al. [18] found that patients treated with PN (HR, 0.45; p = 0.01) or RN (HR, 0.58; p = 0.03) had a significantly decreased CSS than those treated with AS. However, in subgroup analysis of patients aged ≥ 75 years, the instrumental variable analysis showed no statistically significant difference between PN (HR, 0.48; p = 0.1) or RN (HR, 0.57; p = 0.1) and AS in regard of CSS. Additionally, their study also demonstrated the survival benefit of PN over RN. The authors assumed that this result was due to the nephrectomy-induced postoperative renal dysfunction leading to cardiovascular related mortality [19–21].

The role of partial nephrectomy or radical nephrectomy in octogenarian is in debate since the EORTC randomized trial [22] demonstrated that PN was less effective than RN in OS. Tan et al. [23] demonstrated that partial nephrectomy was most effective in age group younger than 75. However, Marchioni et al. [24] demonstrated that PN, compared to RN, in patients aged ≥ 75 was related to lower other cause mortality and was not related to higher cancer specific mortality and 30-days mortality. Ishiyama et al. [25] demonstrated that age was not a significant factor for renal function outcome, negative surgical margin, and perioperative complications in robot-assisted partial nephrectomy in patients ≥ 80 years old. Miller et al. [26] reported through National Cancer database that RN is decreasing in stage 1 renal cell carcinoma in octogenarian group. In current study, we also found RN group tend to have worse survival than PN group, though not significant due to the small number of patients. With a current trend of increasing use of PN, the incidence of PN for small renal masses is increasing [14, 27]. However, in octogenarian group, it is currently still underused. Thus, large population-based study or prospective study including larger number of octogenarian patients underwent PN are warranted to evaluate the role of PN over RN or AS.

The current study has several limitations. At first, even with a large nationwide multi-institutional (KORCC) database, the study population after PSM was still small due to the rarity of octogenarians. Accordingly, the well-known prognostic factors for RCC such as pathologic T stage and Fuhrman grade did not secure statistical significance in PSM cohort. Second, the present study was conducted only with patients who underwent surgery. Thus, the comparative analysis with AS group to evaluate whether the harm of surgery outweighs the marginal survival benefit for some patients could not be performed. In addition, due to the limitations of multi-institution databases, we were not able to provide surgical approach and more comprehensive information on comorbidity, including cardiovascular disease and pulmonary disease. We presented the ECOG performance status, which can estimate the overall patient status, but is insufficient to estimate the physiologic age. A follow-up study with additional information on comorbidity is required.

## Conclusion

The octogenarian RCC group after surgery had comparable survival outcomes compared with younger group after PSM analysis. Thus, for the life expectancy of octogenarian is getting longer, active treatment is considerable in patients with good performance status.

## Supporting information

S1 Fig Flow diagram of the propensity score–matched study cohort. (TIF)

S2 Fig Kaplan-Meier curves for cancer-specific survival (A) and overall survival (B) in patients by age group (octogenarian vs. 60–80 years group) after propensity score matching. (TIF)

S1 Table Baseline characteristics of octogenarians according to the surgery type. (DOCX)

S2 Table Multivariable Cox regression analyses for cancer-specific and other-cause mortality. (DOCX)

S1 File (XLSX)
