# Supplementary figures and images for: Optimal management of renal cell carcinoma in octogenarians: Retrospective analysis using updated Korean Renal Cell Carcinoma (KORCC) database
Source: PLoS One. 2023 Mar 30;18(3):e0283483. doi: 10.1371/journal.pone.0283483 (PMC10062612; doi:10.1371/journal.pone.0283483)

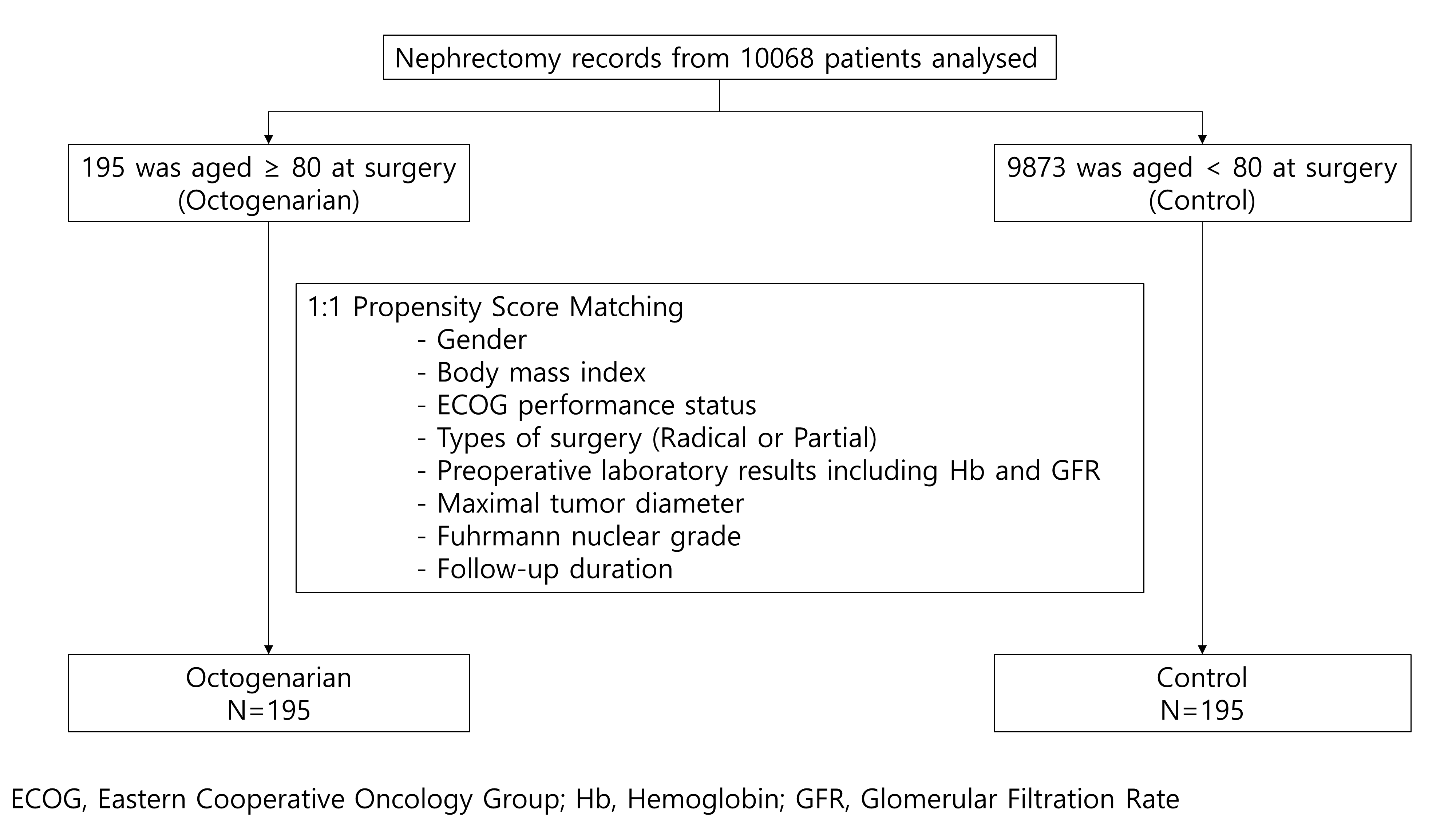

Supplement: S1 Fig — (TIF) [file pone.0283483.s001.tif]

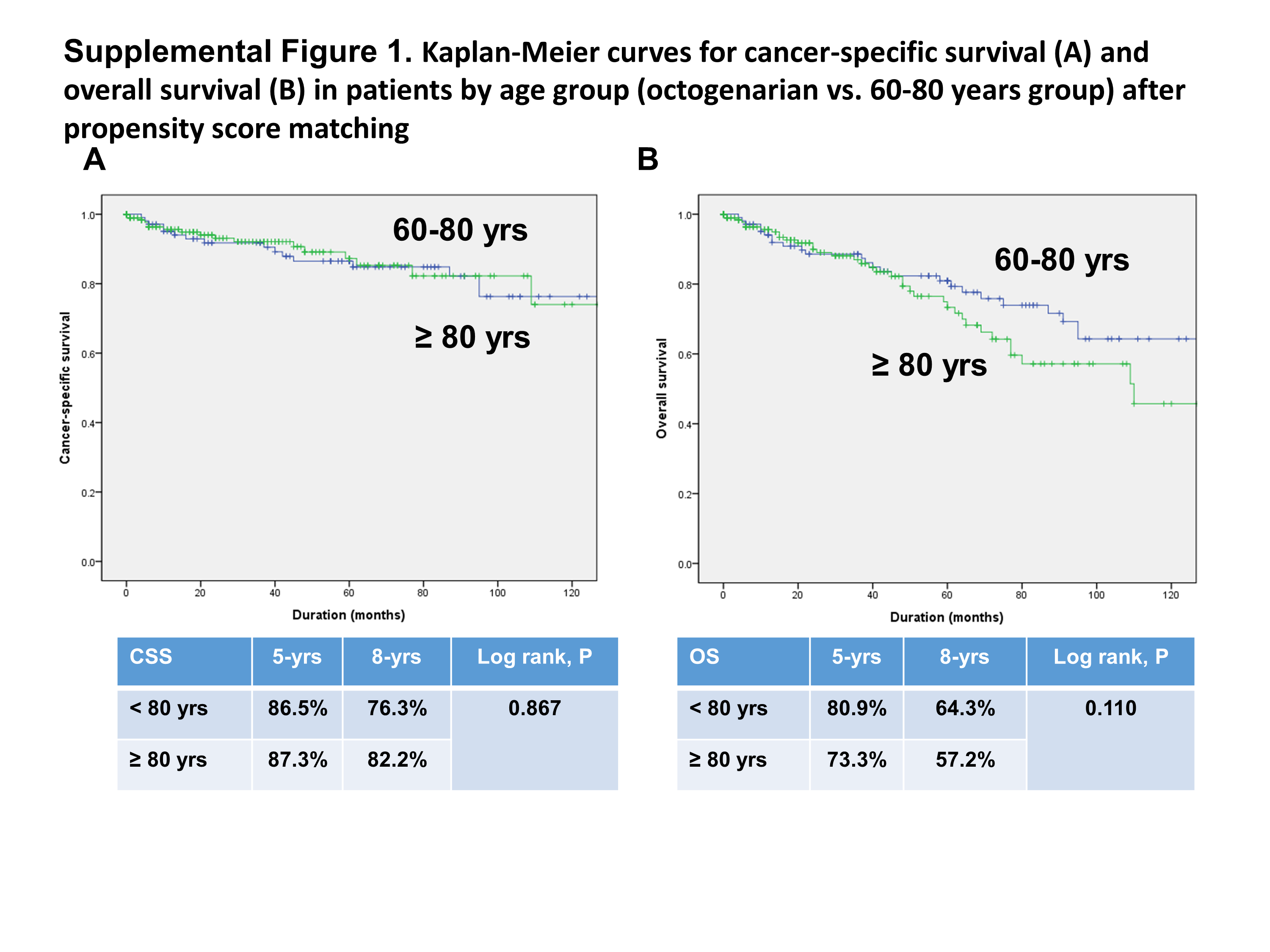

Supplement: S2 Fig — (TIF) [file pone.0283483.s002.tif]
