# Supplementary material for: Optimal management of renal cell carcinoma in octogenarians: Retrospective analysis using updated Korean Renal Cell Carcinoma (KORCC) database
Source: PLoS One. 2023 Mar 30;18(3):e0283483. doi: 10.1371/journal.pone.0283483 (PMC10062612; doi:10.1371/journal.pone.0283483)
Supplement: S1 Table — (DOCX) [file pone.0283483.s003.docx]

**Supplementa1 Table 1. Baseline characteristics of octogenarians according to the surgery type**

| Variables | Radical (N=129) | Partial (N=66) | p |
| --- | --- | --- | --- |
| Age at surgery, yrs, mean (SD) | 82.5 (2.5) | 82.1 (2.2) | 0.226 |
| Gender, n (%) |  |  | 0.398 |
| Male | 70 (54.3) | 40 (60.6) |  |
| Female | 59 (45.7) | 26 (39.4) |  |
| BMI, kg/m^2^, mean (SD) | 23.9 (3.3) | 24.5 (3.0) | 0.238 |
| ECOG performance status, mean (SD) |  |  | 0.163 |
| 0 | 51 (39.5) | 33 (50.0) |  |
| ≥ 1 | 78 (60.5) | 33 (50.0) |  |
| Preoperative laboratory test |  |  |  |
| Hemoglobin, mean (SD) | 12.3 (1.7) | 12.9 (1.4) | 0.024 |
| Serum creatinine, mean (SD) | 1.2 (1.0) | 1.0 (0.4) | 0.175 |
| GFR (MDRD), mean (SD) | 63.4 (21.4) | 72.3 (25.5) | 0.011 |
| EBL, ml, mean (SD) | 343.3 (237.0) | 243.6 (124.0) | 0.063 |
| Operative time, min, mean (SD) | 181.1 (80.2) | 140.1 (45.3) | <0.001 |
| Maximal tumor diameter, mm, mean (SD) | 54.3 (34.6) | 31.1 (15.0) | <0.001 |
| Fuhrman grade, n (%) |  |  | 0.291 |
| G1-2 | 51 (39.5) | 21 (31.8) |  |
| G3-4 | 78 (60.5) | 45 (68.2) |  |
| Pathologic T stage, n (%) |  |  | <0.001 |
| T1-2 | 79 (61.2) | 58 (87.9) |  |
| T3-4 | 50 (38.8) | 8 (12.1) |  |
| Pathologic N stage, n (%) |  |  | 0.426 |
| N0/X | 123 (95.3) | 65 (98.5) |  |
| N1 | 6 (4.7) | 1 (1.5) |  |
| Follow-up, years, mean (SD) | 32.1 (24.6) | 36.5 (32.3) | 0.392 |
| Recurrence, n (%) | 18 (14.0) | 6 (9.1) | 0.328 |
| Cancer-specific mortality, n (%) | 15 (11.6) | 2 (3.0) | 0.059 |
| Overall mortality, n (%) | 33 (25.6) | 6 (9.1) | 0.006 |

SD, standard deviation; BMI, body mass index; ECOG, Eastern Cooperative Oncology Group; GFR, glomerular filtration rate; MDRD, Modification of Diet in Renal Disease Study; EBL, estimated blood loss
