# Supplementary material for: Optimal management of renal cell carcinoma in octogenarians: Retrospective analysis using updated Korean Renal Cell Carcinoma (KORCC) database
Source: PLoS One. 2023 Mar 30;18(3):e0283483. doi: 10.1371/journal.pone.0283483 (PMC10062612; doi:10.1371/journal.pone.0283483)
Supplement: S2 Table — (DOCX) [file pone.0283483.s004.docx]

**Supplemental Table 2. Multivariable Cox regression analyses for cancer-specific and other-cause mortality**

| **Variables** | **Cancer-specific mortality HR (95% CI)** | **P value** | **Other-cause mortality HR (95% CI)** | **P value** |
| --- | --- | --- | --- | --- |
| Age ≥ 80 yrs | 2.385 (1.243-4.580) | 0.009 | 3.153 (2.026-4.909) | <0.001 |
| BMI | 0.926 (0.884-0.969) | 0.001 | 0.916 (0.885-0.949) | <0.001 |
| GFR (MDRD) | 0.993 (0.987-0.999) | 0.015 | 0.987 (0.982-0.991) | <0.001 |
| Gender |  |  |  |  |
| Male | References |  | References |  |
| Female | 0.908 (0.660-1.247) | 0.550 | 0.713 (0.555-0.914) | 0.008 |
| Type of surgery |  |  |  |  |
| Radical nephrectomy | References |  | References |  |
| Partial nephrectomy | 0.340 (0.207-0.559) | <0.001 | 0.626 (0.473-0.827) | 0.001 |
| Maximal tumor size | 1.004 (1.000-1.007) | 0.034 | 1.001 (0.998-1.005) | 0.398 |
| Pathologic T stage |  |  |  |  |
| T1-2 | References |  | References |  |
| T3-4 | 1.886 (1.376-2.585) | <0.001 | 1.768 (1.373-2.276) | <0.001 |
| Pathologic N stage |  |  |  |  |
| N0/X | References |  | References |  |
| N1~~-2~~ | 3.249 (2.185-4.830) | <0.001 | 3.265 (2.273-4.690) | <0.001 |
| Fuhrman grade |  |  |  |  |
| G1-2 | References |  | References |  |
| G3-4 | 2.454 (1.669-3.609) | <0.001 | 1.664 (1.298-2.134) | <0.001 |
| Sarcomatoid differentiation | 2.457 (1.721-3.508) | <0.001 | 2.179 (1.572-3.020) | <0.001 |
| Necrosis | 3.608 (2.470-5.270) | <0.001 | 2.769 (2.084-3.679) | <0.001 |

BMI, body mass index; GFR, glomerular filtration rate; MDRD, The original Modification of Diet in Renal Disease; HR, hazard ratio; CI, confidence interval; HR
